# Modelling Risk to US Military Populations from Stopping Blanket Mandatory Polio Vaccination

**DOI:** 10.1155/2017/7981645

**Published:** 2017-09-14

**Authors:** Colleen Burgess, Andrew Burgess, Kellie McMullen

**Affiliations:** ^1^Ramboll Environ, Inc., Amherst, MA, USA; ^2^MathEcology, LLC, Phoenix, AZ, USA; ^3^Naval Health Research Center, San Diego, CA, USA

## Abstract

**Objectives:**

Transmission of polio poses a threat to military forces when deploying to regions where such viruses are endemic. US-born soldiers generally enter service with immunity resulting from childhood immunization against polio; moreover, new recruits are routinely vaccinated with inactivated poliovirus vaccine (IPV), supplemented based upon deployment circumstances. Given residual protection from childhood vaccination, risk-based vaccination may sufficiently protect troops from polio transmission.

**Methods:**

This analysis employed a mathematical system for polio transmission within military populations interacting with locals in a polio-endemic region to evaluate changes in vaccination policy.

**Results:**

Removal of blanket immunization had no effect on simulated polio incidence among deployed military populations when risk-based immunization was employed; however, when these individuals reintegrated with their base populations, risk of transmission to nondeployed personnel increased by 19%. In the absence of both blanket- and risk-based immunization, transmission to nondeployed populations increased by 25%. The overall number of new infections among nondeployed populations was negligible for both scenarios due to high childhood immunization rates, partial protection against transmission conferred by IPV, and low global disease incidence levels.

**Conclusion:**

Risk-based immunization driven by deployment to polio-endemic regions is sufficient to prevent transmission among both deployed and nondeployed US military populations.

## 1. Introduction

Polio is a viral disease that invades the nervous system and can cause paralysis in a matter of hours, though most poliovirus infections are asymptomatic [1]. There are 3 wild poliovirus (WPV) types, two of which have not been detected globally since 2012 [2]. Polio infection in immunocompetent individuals leads to immunity, although immunity induced by one serotype does not protect against the other two [3]. There are 2 vaccines against polio: inactivated poliovirus vaccine (IPV) and oral poliovirus vaccine (OPV). Successful vaccination with either formulation provides at least partial protection from infection and full disease immunity in approximately 7 days [4].

After IPV immunization, antibodies are produced in the blood in response to the inactivated virus, protecting the individual from disease; however, viral replication in the gut is still possible, with the potential for asymptomatic transmission to the community [5]. IPV-induced antibodies decrease over time, and some adults vaccinated as children may lack a detectable antibody.

In contrast, OPV induces mucosal immunity in the gastrointestinal tract, which is important for community protection [6], and provides long-term immunity against both disease and transmission. Yet, “the oral vaccine often fails in developing countries and in rare cases the vaccine virus itself leads to paralysis” [7]. OPV can also lead to the circulation of a vaccine-induced virus, shed for 1–3 weeks following primary vaccination and easily transmitted within and outside the household. While this shedding is widely accepted as inducing protection or boosting immunity in contacts [3], it can also contribute to the spread of circulating vaccine-derived poliovirus (cVDPV) [6], particularly in settings with weak routine immunization coverage in otherwise polio-free countries [8].

Due to these risks, most developed countries have switched from OPV to IPV [9]. However, for developing countries where polio is endemic or the risk of importation is high, the benefits of OPV outweigh the risks, and, for now, OPV remains the vaccine of choice [3]. The ultimate goal is to eliminate OPV vaccination and switch to IPV-only immunization, thus eliminating the risk of cVDPV altogether. As of November 2016, 173 countries now include IPV in their routine immunizations; the remaining World Health Organization (WHO) member countries are on schedule to introduce IPV by the end of 2016 [10].

Today, WPV remains endemic in 3 countries—Pakistan, Afghanistan, and Nigeria [11]. While WPVs circulate in these areas, “the rest of the world must continue to keep polio vaccination levels very high, due to the risk of outbreaks among susceptible people in polio-free countries” [12]. Response to polio circulation in developing areas such as these generally involves the use of OPV, and “each response round comes with a substantial and uncertain amount of secondary OPV infection” [4], which are of particular risk to deployed US soldiers with incomplete or waning protection against poliomyelitis transmission.

In the United States, children currently receive 3 routine doses of IPV at 2 months, 4 months, and 6–18 months, and a booster at 4–6 years [13]. As a result, US-born soldiers generally enter military service with a high level of immunity to disease, though this may wane over time. The military actively vaccinates all recruits against a number of diseases including polio; “these vaccines are further supplemented, based on occupational and deployment circumstances of the recruits” [14]. However, in countries with insufficient vaccination and/or active viral circulation, contact with local populations puts US warfighters at risk for transmission of both WPV and cVDPV. This is particularly the case since

[although] militaries primarily engage in traditional major combat operations, they are increasingly involved in humanitarian assistance missions. Such missions permit extensive interaction with the local populace and environment, greatly increasing the chance of acquiring locally endemic infectious diseases and necessitating the management of diseases in the local populace that are not traditionally seen in military personnel [15].

Thus, while blanket vaccination of all soldiers may be epidemiologically redundant and cause unnecessary expenditure of resources, risk-based vaccination driven by travel to polio-endemic areas can be appropriate. However, evaluating multiple vaccination strategies once deployment is underway may result in higher-than-necessary disease incidence and cost in an effort to control transmission. In lieu of this, predictive modelling of polio transmission allows for the exploration of vaccination strategies through simulation of multiple scenarios and their outcomes prior to putting troops and mission objectives at risk.

In recent years, published mathematical models for polio transmission have focused primarily on the role of OPV in attaining eradication of the disease. Several mathematical models have also explored vaccine-derived polioviruses [16–18], and additional analyses have addressed the impact of asymptomatic infection [19–22]. Kalkowska et al. [20, 21] explored the possibility of silent transmission of WPV in populations with high IPV coverage, emphasizing that “IPV-based protection alone might not provide sufficient population immunity to prevent poliovirus transmission after an importation” [20] and acknowledging the need to “consider the role of previously-vaccinated or infected individuals (i.e., partially infectible individuals) who remain immune to paralytic disease, but not to reinfection, and their potential participation in silent transmission of the virus” [21]. Most recently, Koopman et al. [22] modelled the interaction between waning immunity and the duration of silent circulation of polio and found that expanding beyond childhood immunization to vaccinate a portion of the adult population could have a significant impact on asymptomatic transmission.

Each of these modelling studies focuses on issues among multigenerational populations with varying levels of immunization coverage and background immunity; none have addressed the unique circumstances affecting polio transmission in highly mobile military populations, which experience conditions that directly affect the spread of disease, both beneficially and detrimentally. US soldiers are vaccinated against polio at recruitment and again prior to deployment to at-risk areas; however, immunization with IPV may leave troops at risk of VDPV or subject to asymptomatic transmission. Quantifying this risk is crucial to evaluating the elimination of mandatory blanket polio vaccination and switching to a solely risk-based vaccination policy.

Mathematical models can be immensely useful in examining the impact of vaccines on disease transmission and are frequently used to inform response policy. For deployed military populations, these models can also evaluate the relative change in transmission risk associated with multiple vaccination scenarios by employing data on specific demographics, epidemiology, and the effects of timing of response. For this analysis, we modified an existing military-specific model system to accommodate polio transmission and vaccination to maximize the achievement of deployed mission objectives, while minimizing the possibility of transmission to troops both abroad and at home.

## 2. Methods

### 2.1. Population Structure

The military population structure for this study was built upon previous analyses [23]. The simulated deployed population consisted of 4 subpopulations defined by interaction with the local population, ranging from negligible to high levels of daily contact. Deployed soldiers were assumed to be posted to a long-standing base within the host country, with well-established water purification and food safety systems to minimize environmental disease transmission. Social mixing was presumed to be mainly within-unit and homogeneous, with between-unit mixing occurring at lower levels.

**Figure 1:**
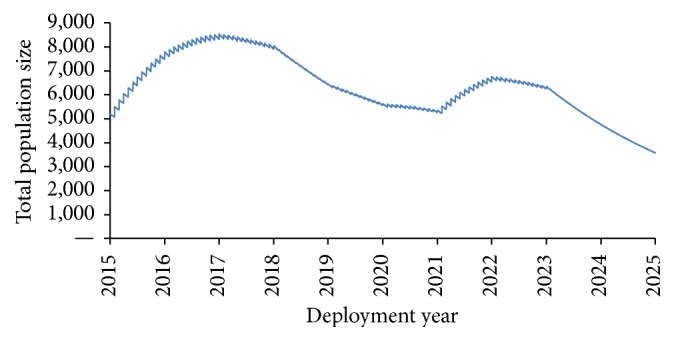
Change in deployed population size over the 10-year duration of the deployment action [23].

The 10-year simulated deployment period began in 2015, with individual soldiers rotating annually. In- and outbound rotation rates (*b*_IN_ and *b*_OUT_) varied over the 10-year period to allow for force increase and decrease (see Figure 1) and a daily casualty rate (*μ*) accounted for removal of individuals for reasons other than rotation or polio-related disease. See Burgess et al. [23] for additional population details.

The local population was modelled without structure and based on the demographic characteristics of Afghanistan as estimated by the United Nations Population Division [24]. Mixing among locals was homogeneous within the simulated geographic region. Local birth and nonpolio death rates varied annually [24], and a polio-specific death rate (*μ*_POLIO_) was applied to infected individuals. OPV immunization of locals was set to historic reported coverage rates (*ρ*) up to 2013 (the most recent year for which data was available at the time of model development) [25] and held constant at the 2013 rate for the remainder of the simulation period.

### 2.2. Transmission Model

**Figure 2:**
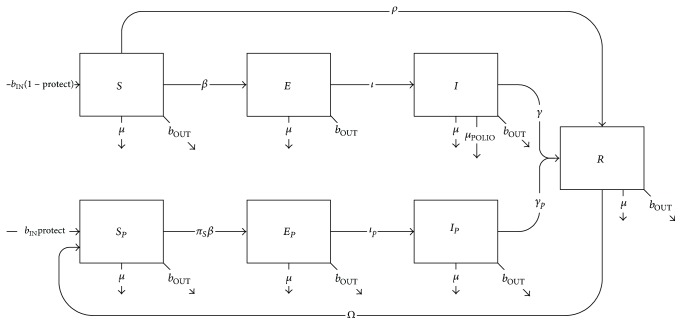
Schematic diagram of polio transmission with both OPV (*ρ*) and IPV (*S*_*p*_) immunization and waning immunity.

Polio transmission was modelled as a modified compartmental Susceptible-Exposed-Infected-Removed model (1) (see Figure 2 and Tables 1 and 2) employing structured ordinary differential equations. Transmission of polio occurred via direct contact between susceptible and infected individuals, with random case importation boosting infection levels stochastically throughout the simulation.

(1) dSidt=bINi1−protectiNi−ρi+βi+μi+bOUTiSi,dSPidt=bINiprotectiNi+ΩRi−πsβi+μi+bOUTiSPi,dEidt=βiSi−ι+μi+bOUTiEi,dEPidt=πsβiSPi−ιp+μi+bOUTiEPi,dIidt=ιEi−γ+μPOLIO+μi+bOUTiIi,dIPidt=ιpEPi−γp+μi+bOUTiIPi,dRidt=ρiSi+γIi+γpIPi−Ω+μi+bOUTiRi,dNidt=bINi−bOUTi−μiNi−μPOLIOIi.

**Figure 3:**
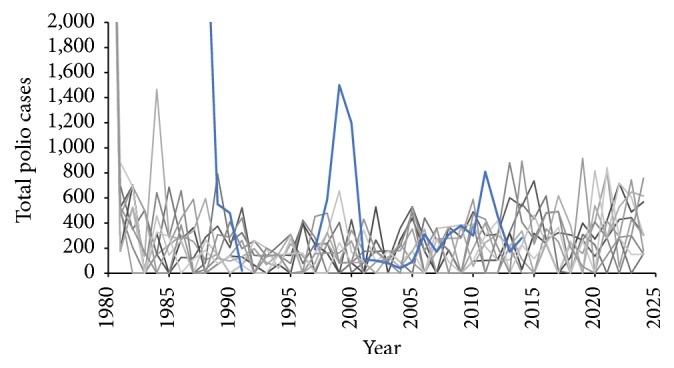
Qualitative comparison between simulated (gray lines) and historical (blue line) polio cases (symptomatic + asymptomatic) for the local population over a sample of 10 random simulations.

To establish endemicity, polio transmission among locals was simulated for a burn-in period of 35 years prior to the arrival of the deployed military population in 2015. Simulated, combined symptomatic and asymptomatic incidence during this burn-in phase was validated against reported paralytic polio cases as recorded by the WHO [34], adjusted to account for the widely accepted 10% proportion of cases that are symptomatic [28]. Validation was performed qualitatively and visually, comparing adjusted historical data to the output of multiple stochastic model iterations under baseline parameter assumptions. Simulated local cases fit well with historical data for 1980–2014 (data missing for 1992–1994 and 1996), in terms of both magnitude and frequency of peaks (Figure 3).

Individuals entered population either via birth (locals) with full susceptibility (*S*) or via inward rotation (military) with partial susceptibility (*S*_*p*_) defined by childhood immunization, blanket immunization upon recruitment, and/or booster immunization prior to deployment (see Scenario Construction). Following an incubation period of 3-4 days (*ι *or* ι*_p_, depending on immune status), exposed individuals (*E*,* E*_*p*_) progressed to infection lasting 27 days (*ϒ*) (range: 27-28 days) for unprotected individuals (*I*) or 9 days (*ϒ*_*p*_) (range: 9–25 days) for partially protected individuals (*I*_*p*_), who were 20% (*π*_*i*_) (range: 20–90%) as infectious as fully unprotected infected persons. Recovered individuals (*R*) possessed immunity to both transmission and disease for a period of 20 years (1/*Ω*) (see discussion of uncertainty), after which only immunity to disease was retained and individuals entered (or reentered) the partially susceptible (*S*_*p*_) class.

Ten percent (symp) of cases in nonimmune, infected individuals presented with symptoms, with the remainder being subclinical; all infections in partially protected individuals were assumed to be asymptomatic. Polio-related mortality affected only symptomatically infected individuals.

Polio transmission was driven by an attack rate (*χ*) of 20 cases per 100,000 population (range: 0.1/1,000,000–20/100,000) with seasonal forcing (seas), allowing for both high- and low-transmission periods within the year. Transmission was further impacted by stochastic case importation with 0.1% probability (importrisk) and amplitude of 1/10th the attack rate (importampl) (see discussion of uncertainty):

(2) seas=1+σcos⁡2πt365,βi=seas∗χ∗∑CijIj+πiIPjNj+import,import=importamplif  rand<importrisk0if  rand≥importrisk,where  rand~U0,1.

### 2.3. Sensitivity and Uncertainty Analyses

Sensitivity and uncertainty analyses were performed on 21 model parameters, with outcomes measured in terms of total symptomatic and asymptomatic polio cases and mean annual incidence among military and local populations.

Total military cases and annual incidence showed direct sensitivity to the relative susceptibility (*π*_*s*_) of partially protected individuals (*S*_*p*_). Since the significant majority of the military population falls into the *S*_*p*_ category, as a result of IPV immunization, it is logical that increased relative susceptibility in these individuals results in more cases overall. In contrast, transmission among locals was dramatically less sensitive to*π*_*s*_, since the only *S*_*p*_ individuals within the local population included those who were previously infected and recovered and for whom enough time had passed that preexisting immunity from recovery waned.

Local annual disease incidence was significantly sensitive to the polio death rate (*μ*_POLIO_); higher mortality results in fewer polio cases, as a result of an overall decrease in individuals participating in transmission. As IPV immunization is assumed to confer at least partial protection, the bulk of polio cases within deployed troops are asymptomatic and, thus, not affected by disease-related death rates.

The duration of the infectious period (1/*ϒ*_*p*_) for partially protected, infected individuals (*I*_*p*_) significantly affected total military cases and annual incidence, reflecting that a shorter infectious period among *I*_*p*_ resulted in lower transmission and, therefore, fewer subsequent polio cases. The impact on local populations was dramatically lower due to the smaller fraction of locals falling within the *I*_*p*_ category.

Local incidence was inversely affected by changes in the duration of OPV- or disease-induced immunity (1/*Ω*), that is, longer-lasting immunity resulted in fewer local cases overall. There was no significant impact on military polio incidence; however, since these individuals were not immunized with OPV, the overall deployment period was not long enough to allow for waning of postrecovery immunity to occur.

The level of residual protection resulting from childhood IPV immunization (childhood) had a moderate impact on polio transmission within military populations in simulations within which no other polio vaccination was administered. In the presence of either blanket or booster vaccination (or both), variation in childhood residual protection had no impact on military polio transmission, since the more recent adult immunization overrode any effects due to vaccination occurring early in life.

Uncertainty in case importation (importrisk and importampl) had a dramatic impact on polio incidence in both military and local populations, overriding even variation in the polio attack rate (*χ*). This mirrors on-the-ground experience with polio eradication efforts. In the absence of new cases entering into an area, adequate routine immunization can halt the local transmission of polio; however, small levels of case importation can spark outbreaks even in immunized regions.

### 2.4. Scenario Construction

To evaluate the hypothesis that residual immunity from childhood vaccination, combined with risk-based deployment vaccination, is sufficient to protect troops from polio transmission, 3 military IPV scenarios were tested:

1. Blanket immunization at recruitment + booster immunization at deployment + residual childhood protection (Scenario 1, baseline).
2. (Blanket immunization terminated in 2015) + booster immunization at deployment + residual childhood protection (Scenario 2).
3. (Blanket immunization terminated in 2015) + residual childhood protection only (Scenario 3).

As of 2013, the US childhood IPV vaccination coverage rate was approximately 93% [25]; that is, 93% of children aged 1–4 years have received 3 routine doses of IPV. The Centers for Disease Control and Prevention's Pink Book [28] indicates that IPV efficacy after 3 doses is approximately 99% (efficacy), and immunity against disease is “probably lifelong”; while there is some debate regarding waning protection, few studies have been performed to evaluate this. To determine residual adult protection resulting from childhood immunization, we developed a waning curve based on data provided by Lapinleimu and Stenvik [35], which describe the change in detectable polio antibodies over time in individuals in Finland where IPV is the only implemented vaccination. From this data, we extrapolated a relationship between detectable antibodies and years since the most recent IPV booster, defined as a function of age. Based on the 2012 Demographics Profile of the Military Community [36], the average age of the active duty force is 28.7 years; when combined with the childhood IPV coverage rate and applied to the waning immunity curve, on average 92% of soldiers still possess detectable antibodies resulting from childhood IPV immunization (childhood).

The childhood IPV coverage rate accounts for both philosophical and medical exemptions to vaccination, including impaired immune status, allergies to vaccine components, or history of vaccine-associated adverse events. Within the 2012-2013 school year, medical exemptions for childhood vaccinations (not specific to polio) ranged from 0.1 to 1.6% (median 0.3%), and nonmedical exemptions ranged from 0.2 to 6.4% (median 1.5%) [37]. While immunization exemptions are allowed for military service members, “noncompliance with immunization requirements may adversely impact deployability, assignment, or international travel,” and “[nonmedical] exemptions may be revoked, in accordance with service-specific policies and procedures, if the individual and/or unit are at imminent risk of exposure to a disease for which an immunization is available” [38].

For this analysis, we assumed that military exemption rates sit at the low-end of the range for childhood vaccination exemptions, yielding an overall military IPV exemption rate (exemption) of 0.3% (range 0.3–8.0%, median 1.8%), with the inclusion of a 75% proportional reduction (dfact) in exemption rate (range 0–100%) for deploying personnel.

Military IPV effective coverage rates for blanket and booster immunization were calculated as follows:

(3) blanket=1−exemption∗efficacy,boost=1−dfact∗exemption∗efficacy.

For Scenarios 2 and 3, blanket immunization upon recruitment was halted in 2015; those individuals recruited prior to 2015 would have relatively recent immunity, while those recruited in 2015 or later would have immunity resulting only from deployment booster immunization (Scenario 2) or residual immunity from childhood immunization (Scenario 3). Thus, for these two scenarios, at any point in time from 2015 through the end of the simulation, there would always be a blend of blanket- and nonblanket-immunized individuals in the deployed population.

As of 2006 [39], accession rates within branches of the US military ranged from 13% for the Air Force to 19% for the Army (accession). For Scenarios 2 and 3, the proportion of service members protected by blanket immunization administered prior to 2015 (*B*(*t*)) was defined as a recursive function diluted by the accession rate:

(4) $$\begin{array}{c} B\left(\;t\;\right)=\left(\;1-\frac{accession}{365}\;\right)*B\left(\;t-1\;\right). \end{array}$$

The net protection level of the military population from prior blanket immunization combined with residual immunity from childhood immunization was, therefore, given as a maximum function, which decayed to no lower than the childhood protection level:

(5) Nt=max⁡Bt∗blanket+1−Bt∗childhood,childhood.

This resulted in a drop in overall protection from 98.7% to 93.0% over the 10-year deployment period.

Scenario-specific parameters are provided in Table 3, and final scenario definitions for deployed and nondeployed military personnel are given in Table 4.

Each scenario was run for 1500 simulations to account for stochasticity in polio case importation. Model outputs were measured as total deployed symptomatic and asymptomatic polio cases and average annual incidence (included both symptomatic and asymptomatic cases) for deployed military and local populations.

## 3. Results

**Figure 4:**
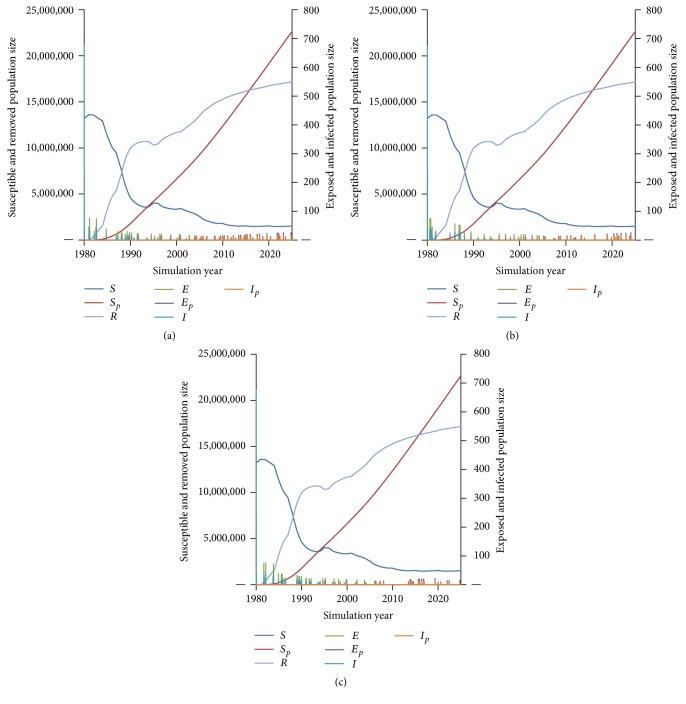
Polio disease dynamics among local populations under military immunization Scenarios (a) 1 (blanket + booster + residual childhood), (b) 2 (booster + residual childhood), and (c) 3 (residual childhood).

For all scenarios, local disease dynamics remained fairly consistent across all simulations, tracking with historical cases prior to 2015, then sustaining low endemicity driven by case importation to the end of the simulation period (Figures 4(a)–4(c)).

**Figure 5:**
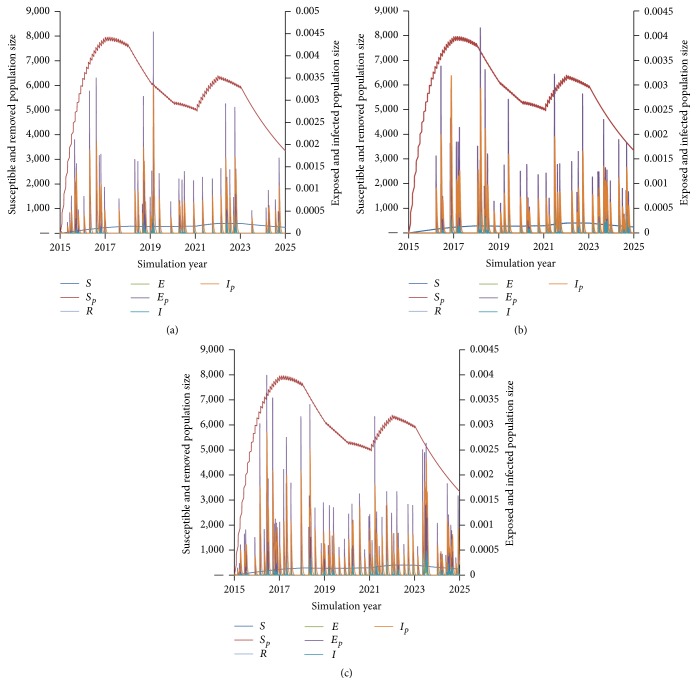
Polio disease dynamics among deployed military populations under military immunization Scenarios (a) 1 (blanket + booster + residual childhood), (b) 2 (booster + residual childhood), and (c) 3 (residual childhood).

Similarly, there was insignificant change in polio dynamics among deployed military populations between Scenarios 1 and 2, with a slight increase in infections under Scenario 3 (Figures 5(a)–5(c)).

**Figure 6:**
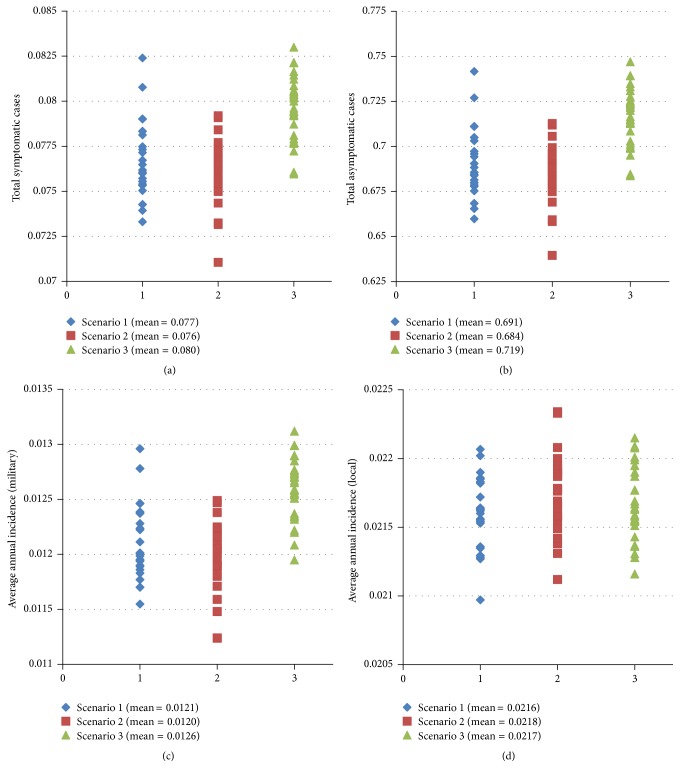
Distribution over 1500 simulations per scenario of (a) total symptomatic polio cases in deployed military populations; (b) total asymptomatic polio cases in deployed military populations; (c) average annual polio incidence in deployed military populations; and (d) average annual polio incidence in local populations under 3 military immunization scenarios.

Stochasticity associated with case importation caused variation between simulation results for total cases and average annual polio incidence for all 3 scenarios (Figures 6(a)–6(d)). This variation was relatively small; however, and data points were generally well-clustered around mean values.

Dropping blanket immunization but maintaining predeployment booster immunization had negligible effect on simulated deployed military cases and incidence. Dropping both blanket and predeployment immunizations yielded a 5% increase in polio cases and annual incidence among deployed populations over the baseline scenario of blanket immunization (Table 5). Local annual incidence was not significantly affected by changes in military immunization strategies, with any variation resulting only from model stochasticity, which indicated that transmission from military to local populations was not an important issue at these levels of military protection.

The total number of symptomatic and asymptomatic polio cases in the deployed military population remained less than one for all 3 immunization scenarios, though fractional cases were still utilized in the calculation of incidence rates. Though frequently undetectable in the field, asymptomatic cases were included in the case-count and incidence calculations to provide a measure of the potential for silent transmission.

For nondeployed personnel, dropping blanket immunization resulted in a decrease in polio disease protection from 99% to 93%. Since IPV vaccination confers full protection from disease but only partial (model assumption: 20%) protection from transmission, this yielded an increase in overall susceptibility to transmission among the nondeployed population from 21% to 26%.

Combining nondeployed susceptibility levels with the average annual polio incidence among deployed populations for each scenario allowed for estimation of the risk of new polio infections within nondeployed personnel due to mixing with infected soldiers returning from deployment. For the blanket immunization scenario (Scenario 1), the risk of new polio infections resulting from reintegrating infected soldiers was predicted to be 0.000504/1,000,000. For Scenario 2—where blanket immunization was terminated but predeployment booster was still employed—the simulated risk of new polio infections increased from 0.000504/1,000,000 to 0.000624/1,000,000 after 10 years without blanket vaccination. For Scenario 3—where both blanket and booster immunizations were terminated—simulated risk of new polio infections among nondeployed personnel increased from 0.000546/1,000,000 to 0.000676/1,000,000 (Table 6) over the same 10-year period.

## 4. Discussion and Conclusions

Mathematical models can help guide preventive medicine policy, resulting in healthier and protected populations. This analysis employed a mathematical model for the transmission of polio within deployed military populations interacting with local populations in an endemic setting. Results from model simulations described the potential benefits of protecting these troops via routine blanket immunization, predeployment booster immunization, and residual protection resulting from childhood vaccination.

In the absence of blanket immunization on recruitment, immunity to polio disease among* nondeployed* personnel defaults gradually to residual protection resulting from childhood immunization, and the percentage of this population susceptible to transmission increases. Although the removal of simulated blanket immunization had no noticeable effect on polio incidence among* deployed* personnel subject to predeployment booster immunization, the risk of transmission to nondeployed personnel mixing with deployed soldiers reintegrating with base populations increased by 19%. In the absence of predeployment booster immunization, risk of transmission to nondeployed populations increased by 25% over the baseline scenario.

Though the increased percentage in transmission resulting from dropping blanket immunization was nonzero, the overall risk of new infections among both deployed and nondeployed service members was extremely low, resulting from the combination of high US childhood immunization coverage rates, conferment of partial protection against polio transmission by IPV, and low disease incidence levels globally. At this range of risk, the likelihood of importation of polio cases among deployed soldiers, and subsequent spread to their nondeployed counterparts, is exceptionally small even in the absence of blanket immunization.

Given preexisting protection resulting from routine childhood vaccination, predeployment booster of service members driven by travel to polio-endemic regions is sufficient to prevent additional transmission among both deployed and nondeployed populations based on these results. Blanket mandatory polio vaccination of Department of Defense service members appears to be epidemiologically redundant, and dropping this routine immunization will not adversely affect troop readiness or mission objectives.

## Figures and Tables

**Table 1:** Variable definitions for polio transmission model.

Variable	Definition
*S*_*i*_	Number of unprotected susceptible individuals in subpopulation *i*
*S*_*Pi*_	Number of partially protected susceptible individuals in subpopulation *i*
*E*_*i*_	Number of unprotected exposed individuals in subpopulation *i*
*E*_*Pi*_	Number of partially protected exposed individuals in subpopulation *i*
*I*_*i*_	Number of unprotected infected individuals in subpopulation *i*
*I*_*Pi*_	Number of partially protected infected individuals in subpopulation *i*
*R*_*i*_	Number of recovered or removed individuals in subpopulation *i*
*N*_*i*_	Total population size in subpopulation *i*

**Table 2:** Parameter definitions for polio transmission model for military (MIL) and local (LOC) populations.

Parameter	Definition	Value (range)	Source
*b*_IN*i*_	Daily inbound rotation rate (MIL)/birth rate (LOC) for subpopulation *i*	[Array]	
*b*_OUT*i*_	Daily outbound rotation rate (MIL)/background death rate (LOC) for subpopulation *i*	[Array]	
*µ*_*i*_	Daily background casualty rate for subpopulation *i* (MIL)	[Array]	
protect_*i*_	Daily proportion of inbound population with preexisting partial protection for subpopulation *i* (MIL)	[Array]	
*ρ*_*i*_	Daily (OPV) vaccination rate for subpopulation *i* (LOC)	[Array]	
*β*_*i*_	Effective polio transmission rate for subpopulation *i*	[Function]	
*π*_*s*_	Relative susceptibility of *S*_*p*_ individuals	0.2 (0.2–0.9)	[26]
1/*ι*	Duration of latent period for unprotected individuals	3 d (3-4 d)	[26, 27]
1/*ι*_*p*_	Duration of latent period for partially protected individuals	4 d (3-4 d)	[26, 27]
1/*γ*	Duration of infectious period for unprotected individuals	27 d (27-28 d)	[26]
1/*γ*_*p*_	Duration of infectious period for partially protected individuals	9 d (9–25 d)	[26]
*μ*_POLIO_	Polio mortality rate	0.22 (0.02–0.30)	[28]
*Ω*	1/duration of OPV or disease-induced immunity	1/(365*∗*20)	(See text)
seas	Seasonal variation in polio transmission	[Function]	
*σ*	Proportional change in polio transmission due to seasonality	0.15	[21]
*χ*	Polio attack rate	20/100,000 (0.1/1,000,000–6.8/100,000)	[29–32]
*C*_*ij*_	Daily contact rate between subpopulations *i* and *j*	[Array]	[23]
*π*_*i*_	Relative infectiousness of *I*_*p*_ individuals	0.2 (0.2–0.9)	[26, 33]
symp	Proportion of unprotected polio cases that are symptomatic	0.1	[28]
importrisk	Probability of polio case importation from outside population	0.001	(See text)
importampl	Amplitude of polio case importation from outside population	2/100,000	(See text)

**Table 3:** Parameter definitions for scenario calculations.

Parameter	Definition	Value (range)	Source
efficacy	IPV vaccine efficacy (MIL)	99% (50–100%)	[28]
childhood	Residual protection level from childhood IPV vaccination	0.92 (0.0–1.0)	Calculated from [25, 35]
exemption	Overall military vaccination exemption rate (medical + administrative)	0.003 (0.003–0.08)	Calculated from [37]
dfact	Proportional reduction in exemption rate for deployed personnel (versus nondeployed)	0.75 (0.0–1.0)	(Estimated)
blanket	IPV blanket vaccine coverage (when implemented) for all military personnel upon accession	(Function)	
boost	IPV boost vaccine coverage (when implemented) for deploying personnel	(Function)	
accession	Military accession rate	0.19 (0.13–0.19)	[39]
*B*	Proportion of military population covered by blanket vaccination prior to 2015	(Function)	
*N*	Overall protection of military population from blanket vaccination prior to 2015 and residual childhood immunity	(Function)	

**Table 4:** Scenario definitions for deployed and nondeployed military personnel.

	Protection levels (protect)	
	Deployed personnel	Nondeployed personnel
Scenario 1 (baseline)	blanket	blanket
Scenario 2 (booster)	boost	*N*(*t*)
Scenario 3 (childhood)	*N*(*t*)	*N*(*t*)

**Table 5:** Median total polio cases and average annual incidence with percentage of change over baseline for deployed military and local populations under 3 military immunization scenarios.

Scenario	Total cases (military)		Average annual incidence	
	Symptomatic	Asymptomatic	Military	Local
1	0.076	0.687	0.012/1000	0.022/1000
2	0.076 (+0%)	0.687 (+0%)	0.012/1000 (+0.2%)	0.022/1000 (+0.8%)
3	0.080 (+5%)	0.721 (+5%)	0.013/1000 (+5.4%)	0.022/1000 (+0.1%)

**Table 6:** Modelled risk of new polio infections among nondeployed service members as a result of infected soldiers reintegrating upon return from deployment.

Scenario	% protected from disease (deployed)	Average annual incidence (nondeployed)	% protected from disease (nondeployed)	% susceptible to transmission (nondeployed)	Risk of new infections (nondeployed)
1	99%	1.2/100,000	99%	21%	0.000504/1,000,000
2	99%	1.2/100,000	99% *↘* 93%	21% *↗* 26%	0.000504/1,000,000 *↗* 0.000624/1,000,000
3	99% *↘* 93%	1.3/100,000	99% *↘* 93%	21% *↗* 26%	0.000546/1,000,000 *↗* 0.000676/1,000,000
